# Development of an intervention to manage benzodiazepine dependence and high-risk use in the context of escalating drug related deaths in Scotland: an application of the MRC framework

**DOI:** 10.1186/s12913-023-10201-7

**Published:** 2023-11-04

**Authors:** Karen Berry, Catriona Matheson, Joe Schofield, Joshua Dumbrell, Tessa Parkes, Duncan Hill, Mary Kilonzo, Graeme MacLennan, Duncan Stewart, Trina Ritchie, Michael Turner

**Affiliations:** 1 University of Stirling, Stirling, Scotland; 2 The University of Edinburgh, Edinburgh, Scotland; 3 NHS Lanarkshire, Lanarkshire, Scotland; 4 University of Aberdeen, Aberdeen, Scotland; 5 NHS Lothian, Edinburgh, Scotland; 6 NHS Greater Glasgow and Clyde, Glasgow, Scotland; 7 NHS Grampian, Aberdeen, Scotland

**Keywords:** Intervention development, Co-production, Drug related deaths, Benzodiazepines, Opiates, PWLE, Polydrug use

## Abstract

**Background:**

Scotland has the highest rate of drug related deaths (DRD) in Europe. These are deaths in people who use drugs such as heroin, cocaine, benzodiazepines and gabapentinoids. It is a feature of deaths in Scotland that people use combinations of drugs which increases the chance of a DRD. Many deaths involve ‘street’ benzodiazepines, especially a drug called etizolam. Many of the ‘street’ benzodiazepines are not licensed in the UK so come from illegal sources. People who use opiates can be prescribed a safer replacement medication (e.g., methadone). While guidance on management of benzodiazepines use highlights that there is little evidence to support replacement prescribing, practice and evidence are emerging.

**Aim:**

To develop an intervention to address ‘street’ benzodiazepines use in people who also use opiates.

**Methods:**

The MRC Framework for Complex Interventions was used to inform research design. Co-production of the intervention was achieved through three online workshops with clinicians, academics working in the area of substance use, and people with lived experience (PWLE). Each workshop was followed by a PWLE group meeting. Outputs from workshops were discussed and refined by the PWLE group and then further explored at the next workshop.

**Results:**

After these six sessions, a finalised logic model for the intervention was successfully achieved that was acceptable to clinicians and PWLE. Key components of the intervention were: prescribing of diazepam; anxiety management, sleep, and pain; and harm reduction resources (locked box and a range of tips), personal safety conversations, as well as a virtual learning environment.

**Conclusion:**

A co-produced intervention was developed for next stage clinical feasibility testing.

**Supplementary Information:**

The online version contains supplementary material available at 10.1186/s12913-023-10201-7.

## Background

The problematic use of benzodiazepines contributes to harms and mortality among people who use drugs. People who use drugs can consume ‘megadoses’ of benzodiazepines, usually in combination with other drugs, which combine to increase the risk of harm [1, 2]. Etizolam, a benzodiazepine-like drug not licensed in the UK, has been widely implicated in the rise of drug related deaths (DRD). In 2021, of the 1330 DRD in Scotland, ‘street’ benzodiazepines were implicated in 842 deaths (63%), with benzodiazepines in general being implicated in 69% of DRD in the same year [3]. ‘Street’ benzodiazepines refers to those benzodiazepines that have been obtained illegally and/or are not licenced for prescribing in the UK, for the reminder of the paper these will be referred to as benzodiazepines only. Several Scottish Government strategy documents have expressed concern regarding the increased prevalence of benzodiazepines-type drugs [4].

UK Clinical guidelines recommend short-term prescribing of benzodiazepines for anxiety and panic disorders but these are often prescribed for much longer [5]. Adverse effects include impaired coordination, amnesia, cognitive impairment, and dependence [1]. UK Guidelines on the clinical management of problem drug use and dependence state that, in relation to an individual being dependent on benzodiazepines, pharmacological interventions may have a role but acknowledge that there is little evidence to support long term substitute prescribing [6]. These guidelines acknowledge that optimal dose and speed of tapering is not known [6]. Benzodiazepine withdrawal can be unpleasant and prolonged if dependence is long established. Abrupt cessation can cause seizures. UK guidelines on clinical management recommend deprescribing for people who use drugs receiving opiate replacement therapy (ORT) who also use benzodiazepines [6]. Deprescribing is a process of gradual reduction (tapering)_ of the daily dose over a period of time. The Clinical Guidelines suggests three months as a time frame. Similarly, Public Health England (PHE) recommends deprescribing but, importantly, notes that inappropriately limiting prescribed supplies can have adverse physical, emotional and social effects if people are dependent on them [7]. Research evidence has previously focussed on managing benzodiazepine dependence through gradual reduction of the benzodiazepines. A 2009 meta-analysis indicated that gradual reduction with psychosocial intervention gave the most effective results. Authors concluded that there was insufficient evidence to support a substitution approach at that time [8]. Similarly, a 2018 Cochrane review concluded “it is not possible to draw firm conclusions regarding pharmacological interventions to facilitate benzodiazepine discontinuation in chronic users [9]. However, a recent review found a number of non-randomised studies that investigated the safety and patient-centred outcomes of co-prescribing ORT and benzodiazepines [10]. Whilst all-cause mortality was increased in 4 of 5 studies, there were other considerations such as improved treatment retention in those prescribed a benzodiazepine [11, 12].

Clinical commentators, such as Lader, have concluded that benzodiazepine maintenance prescribing, following a harm reduction model, could be an appropriate strategy [13]. Darke and Farrell devised a scale to assess the suitability of drug groups for substitution treatment and concluded that, while the case for benzodiazepines was not as strong as for nicotine or opiates, it could be suitable [14]. A systematic review of general practitioner prescribing found that many have successfully prescribed diazepam to patients receiving ORT for extended periods of time [15].

The Scottish situation is slightly different from other parts of the world (and UK) because escalating drug deaths are strongly associated with increasing use of benzodiazepines in polydrug combinations [16]. Data supplied by National Records of Scotland illustrate that the increase in DRD is driven by a combination of etizolam with heroin and/or methadone, often in combination with cocaine and gabapentinoids [17]. Some Scottish clinicians have been reluctant to consider benzodiazepine maintenance prescribing due to the lack of evidence of its efficacy. However, given the unique situation and the risk of exposure to benzodiazepines, a harm reduction-based intervention that incorporates a prescribing element was considered worthy of exploration. Anecdotally it was suggested that some benzodiazepine prescribing was being undertaken by clinicians in Scotland. However, it was not clear whether this was following clinical deprescribing guidance described above or whether some clinicians were open to a more relaxed prescribing regime which could be considered as maintenance or a slow reduction.

**Fig. 1:**
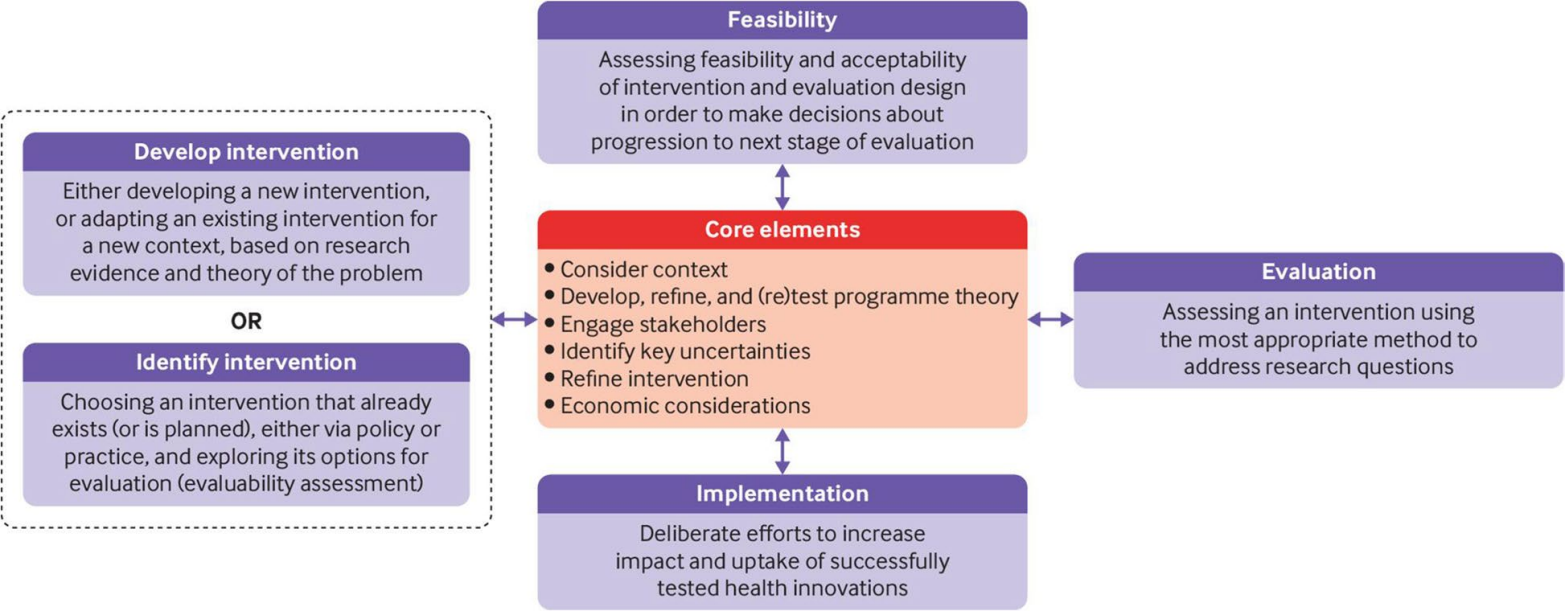
MRC framework for complex interventions [20]

Following the revised MRC Framework for Complex Interventions (Fig. 1), two pieces of preparatory work were carried out by the research team, qualitative interviews with people who use street benzodiazepines and a survey of prescribers treating people who use drugs in Scotland. The qualitative piece of work focussed on exploring this group’s motivations for using benzodiazepines and their views on possible treatments. This group were aware of the risks of benzodiazepines but avoiding them was difficult due their low cost and ease of availability. The survey focussed on clinicians’ current benzodiazepine prescribing practices; 67% (*n* = 55) reported currently prescribing benzodiazepines to people with benzodiazepine dependence who also use opiates. Of the 17 who reported not currently prescribing benzodiazepines 11 said that would be willing to do so in the future. Of that group some stated that they would only consider prescribing benzodiazepines if there was clear and robust clinical evidence of benefit and harm reduction [18, 19].

The intervention development reported in this paper covers work package 1 of a larger study funded by the Chief Scientists Office for Scotland. The focus of work package 2 was to conduct a feasibility study of the intervention developed in work package 1 to assess its acceptability to clinicians and patients and to explore the feasibility of recruiting and retaining patients in such an intervention^1^ and testing the use of appropriate outcome measures.

This paper describes only work package 1, the development of a theory informed, co-designed intervention for the clinical management of street benzodiazepines use in those on ORT using the revised MRC framework. The population of interest was drug users being prescribed ORT with a co-existing benzodiazepine dependence. The revised framework describes an emphasis on considering and understanding both the systems and context of intervention development, and the importance of ensuring built-in flexibility, in order to be effective across a range of settings.

## Methods

The study used a target population-centred approach to intervention development [21] in which the views of those participating in the intervention were central and involved throughout. The intervention development and following feasibility study also followed the revised MRC Framework for Complex Interventions [22] and guidance on developing complex interventions [23]. Table 1 shows the combined recommended steps from both approaches and how this was achieved in this study. This intervention itself was informed by the theoretical perspective of harm reduction; a pragmatic approach to drug use in which the aim of treatment and care is to reduce the harm to people who use drugs [24]. The use of benzodiazepines is known to increase the risk of overdose or a DRD (due to the polydrug combination of sedatives) and, therefore, a pragmatic, non-judgemental approach to safely reduce benzodiazepines use that puts the person who uses these drugs at the centre of care decision-making, was required.

**Table 1:** Aspects of our development process informed by intervention development taxonomy

Domains	Actions (numbering relates to the taxonomy of intervention [21] development approaches	How this was achieved
Planning	2. Establishing a group or set of groups to guide the development process, thinking about engagement of relevant stakeholders such as the public, patients, practitioners, and policy makers	Two bespoke groups were set up to take part in the intervention development process 1. Intervention development group membership of 20 including, academics working in the area of substance use, NHS substance use consultants, clinical psychologists with topic expertise, community psychiatric nurses, GP’s and a person with lived experience 2. (PWLE) Group A group made of seven members, men and women who were currently using or had previous experience of using benzodiazepines This group was recruited by our community researcher JD using their contacts in this population. The group was made up of seven members, men and women who were currently using or had previous experience of using street benzodiazepines and opiates These two groups participated in the intervention development process. The separate groups were established so that members of the PWLE group were represented in the intervention development group but had their own forum where it was felt they would feel more comfortable to talk openly about their experiences and the intervention itself
	3. Understand the problems or issues to be addressed	Preparatory work was undertaken to explore the issues in this area including interviews with patients currently using benzodiazepines and a survey with clinicians around benzodiazepines prescribing in Scotland. Combined with DRD statistics [3] this illustrated the issues around the importance of addressing benzodiazepine use and its increasing links to DRD in Scotland. Opiates and benzodiazepines are increasingly seen linked in DRD in Scotland and as such this group was identified as the target group for the planned intervention
	5. Identify possible ways of making changes to address the problems. This involves identifying what needs to change, how to bring about this change and what might need to change at individual, interpersonal, organisational, community or societal levels	Changes in benzodiazepine prescribing in the community setting in Scotland was identified as an area which could have contributed to an increase in the use of street benzodiazepines. The reduction in GP benzodiazepine prescribing because of initiatives to reduce GP prescribing through guidance and training, was seen as a driving factor in this increase in street benzodiazepine use. With polydrug use including opiate and benzodiazepine use linked to increases in DRD there needs to be research to understand this better Individual • PWLE need an alternative to seeking street benzodiazepines Interpersonal • Understanding PWLE’s reasons for using street benzodiazepines, how could these be addressed in an intervention Organisational • Consideration around the prescribing of benzodiazepines in those also being prescribed ORT • Access to psychological services for PWLE who are receiving ORT
	7. Consider real-world issues about cost and delivery of any intervention at this early stage to reduce the risk of implementation failure at a later stage	The research team considered who currently provided community support to PWLE as they would be best placed and the least expensive to upskill to deliver the developed intervention. This was designed to allow the intervention to be developed within the existing care and harnessing existing expertise in this area. Community Psychiatric Nurses (CPNs) were identified as being well positioned to be the main mode of intervention delivery Also considered was the additional cost to services of prescribing diazepam to this group, the additional nursing time required to deliver the psychosocial aspects of the intervention and the possibility of an increased workload for addiction services relating to re-engagement of patients seeking this intervention
	8. Consider whether it is worthwhile continuing with the process of developing the intervention	Early discussion with the broad membership of the intervention development and PWLE groups
Designing	9. Generate ideas about solutions and components and features of an intervention	Preparatory work indicated that the intervention should include both prescribing and psychosocial components and would be delivered by existing staff within the current treatment system to manage costs associated with delivering the intervention
	10. Re-visit decisions about where to intervene. This can involve consideration of the different levels at which to intervene, and the wider system in which the intervention will operate	We regularly revisited the practicalities of delivering the intervention as originally planned for example the length of consultation time and frequency of consultations and what could practically be covered during a consultation. Consideration was given to the wider context of intervention delivery as well as who would be best able to deliver the intervention successfully without the need for a new care pathway
Creating	13. Make prototypes or mock-ups of the intervention, where relevant	We developed a draft of the intervention which was disseminated to both the intervention development group and the PWLE group for comment. These comments were then used to refine and finalise the intervention
Documenting	17. Document the intervention, describing the intervention so others can use it and offer instructions on how to train practitioners delivering the intervention on how to implement the intervention	The intervention was documented using the template for intervention description and replication (TIDieR) guidelines [25] ( REF _ REF _Ref1 REF _ REF _ REFAppendix 1. TIDieR for Benzodiazepine Intervention Development Study Furthermore, feedback through semi-structured interviews with clinical delivery staff as well as patients enabled the identification of mediators and barriers
Planning full evaluation	18. Develop the objectives of the outcomes and process evaluation This includes determining how outcomes and mediators of change can be measured, developing measures, specifying evaluation design, recruitment and considering feasibility of a full RCT	The starting point for the intervention development in the workshops was an outline logic model which showed existing knowledge and what was needed from the group during the development process One aspect related to the outcomes which were deemed as important for the feasibility testing of the developed intervention. Some of these had been identified previously and this was supplemented during the development process itself These outcomes are currently being used in the feasibility testing of the intervention and will be amended as needed for a possible full RCT in the future Mediators of change identified in the feasibility will be assessed formally as part of a process evaluation in a full trial

To reduce risks to participants associated with the COVID-19 pandemic and social restrictions at the time, online meetings and workshops were held on either Teams or Zoom platforms. We convened two separate groups to participate in the intervention development process. Firstly, an intervention development group which consisted of members of an existing benzodiazepine management research interest group including clinicians, practitioners and academics, people with living experience of benzodiazepine use, and experts in intervention development plus one member of the PWLE group for oversight of the full process. There were 20 members of this group, each of whom attended at least one of the intervention development workshops. The second group consisted of people with living and previous experience of benzodiazepine use; these seven participants (5 women, 2 men) made up the PWLE group. At least five members of this group were present at each PWLE meeting. A series of six meetings were conducted: three online workshops with clinicians, academics working in substance use, and three with people with lived experience (PWLE). Each workshop was followed by a PWLE group meeting. Outputs from workshops were discussed and refined by the PWLE group and then further explored at the next workshop.

The three intervention development workshops and the three PWLE meetings followed the same topics and structure, some variation related to each groups focus/perspective, i.e., only covering eligibility criteria with clinical/academic members of the team and discussing use/past use of benzodiazepines with the PWLE group Table 2.

**Table 2:** Intervention development workshop plan

Workshop 1	Introduction and expectations of the group
	Introduction to the outline logic model
	Discussion on the context of the proposed intervention
Workshop 2	Discussion on the possible intervention outcomes
	Eligibility criteria
	Possible intervention components – prescribing and psychosocial
Workshop 3	Discussed feedback from PWLE meeting two on proposed intervention components
	Practicalities of delivering the intervention in proposed feasibility study sites

Members of both the intervention development and PWLE groups were provided with full results from the preparatory work which had been carried out including the survey and interviews with people who were using benzodiazepines [18, 19]. These were reviewed and discussed at the first meeting. Intervention workshop facilitation was undertaken by CM (PI) and KB (RF) and the PWLE meetings were facilitated by KB (RF) and JD (PWLE lead).

## Results

### Workshop 1

As a starting point, an outline logic model developed by the research team, was introduced to both groups in Workshop 1 to help clarify what areas we would be looking to establish through the intervention development process. The area of the logic model that was addressed in Workshop 1 focussed on the context of the intervention. We were able to draw together the findings from the context setting exercise that we carried out with both the intervention development and PWLE groups. This process enabled us to identify the influential factors which could affect the development of the intervention as well as its potential outcomes for example clinician ‘buy-in’ to the process and potential implications for prescribing practice. This process additionally allowed us to think about what aspects of the immediate environment we would be able to influence with the intervention and what aspects would be beyond its scope.

The two groups, the intervention development group and the PWLE group came to this process from vastly different perspectives. The intervention development group from a position of delivering care within addiction services and those in the PWLE group from the perspective of having experienced receiving care from those same services. Due to their potentially opposing perspectives, there were areas that the intervention development group identified as being able to control but that the PWLE group saw as being out with their control. These included prescribing practices, communication with services, including CPNs and GPs, accessing psychosocial support, staff, and training. Table 3 outlines the issues highlighted by the PWLE group in relation to benzodiazepine prescribing and reduction in currently available services. Both the intervention development group and the PWLE group identified high risk benzodiazepine use including ‘megadosing’ as an issue among this patient group. These findings corroborated the initial idea around developing an intervention with a prescribing element and an element of psychosocial support. In discussing the issues of context in both groups we were able to see what we would be unable to influence with this intervention, for example access to existing psychological services for patients still using benzodiazepines. However, we would be able to provide enhanced psychosocial care for this group as part of the intervention through additional training for those delivering the intervention.

**Table 3:** Main issues around benzodiazepine prescribing raised by the PWLE group

Main areas of concern from the PWLE Group		
Benzodiazepine prescribing	Fear around reducing benzodiazepines	Lack of psychosocial support and communication
• Prescribing has been reduced but there doesn’t seem to be a ‘plan of action’ on how to do this or alternatives • No support on reducing/stopping benzodiazepine use (prescribed or street) • Postcode lottery around benzodiazepine prescribing-didn’t want to move to a different GP in case they lost their prescription • Left on the same ‘maintenance dose’ for prolonged periods of time with no next steps discussed • Cutting off prescriptions as they are believed to be risky or dangerous is ‘ridiculous’ due to the availability	• Fear of raising the subject of talking to care providers about reducing for fear of having their prescription stopped immediately • Fear of starting these conversations as there is little/no support for stopping benzodiazepine use • ‘Scared if you reach out, scared if you don’t’ • Fear of telling GP/addiction services of any illicit drug use in case they lose their current prescription • Fear around seeking treatment/support in the first place because of anonymity issues, once they have approached services their drug use is recorded, worries around stigma that could arise in the future as a result	• Feelings that doctors are ‘closed door’, not willing to discuss options • People are given reduction plans but no trauma work, psychosocial support while doses are reduced • Trauma is rarely dealt with – offered prescriptions which are slowly reduced over time, but because the original trauma and/or anxiety was never handled, it often results in further illicit use • Psychosocial support is required to help these individuals. There is poor communication between prescribers/service providers and people who use drugs

In addition, these discussions identified three areas of commonality in the findings from both groups, the need for better access to psychosocial support for patients still using benzodiazepines, better communication between services and between services and patients themselves.

### Workshop 2

This workshop began the process of identifying possible intervention components within the elements of prescribing and psychosocial support. In this workshop the group was split into three breakout rooms to discuss different aspects of the intervention. The first break out group, made up of clinicians, focussed on the eligibility criteria around prescribing benzodiazepines to patients already prescribed ORT. These eligibility criteria allowed them the basis for what would be prescribed to participants and on what basis. The two other breakout groups worked on proposing possible components of the psychosocial aspect of the intervention. These discussions identified that there should also be a third component of the intervention looking at harm reduction.

Following this intervention development workshop, a draft intervention was developed which was then circulated to the PWLE group ahead of their next meeting. At the second PWLE meeting we facilitated a discussion with the group on the draft intervention so that we could find out if there were any gaps from their perspective or anything that they felt could be done differently. They were positive about the draft intervention but had several questions relating the prescribing aspect of the intervention and what would happen to feasibility participants at the end of the intervention period. Their discussion focussed heavily on the harm reduction aspect of the intervention and several ideas on how this could be delivered including a buddy system, text messaging, drug checking service, and an online platform for participants. Within the constraints of the study, we chose to develop an online platform containing harm reduction information as well as information relating to each aspect of the intervention including trauma, sleep hygiene, anxiety, and a discussion board for peer support.

### Workshop 3

The final intervention development group workshop focussed on incorporating the PWLE intervention feedback to develop a finalised intervention. Following the final workshop this final intervention was disseminated to both groups for approval. The intervention development group also worked on the practicalities of delivering the developed intervention across our three feasibility study sites.

In our third and final PWLE workshop we focussed on using their experience to begin the process of developing the online platform in response to their previous suggestion. We continued to work with the PWLE group in the development of the online platform beyond the specified intervention development process.

**Fig. 2:**
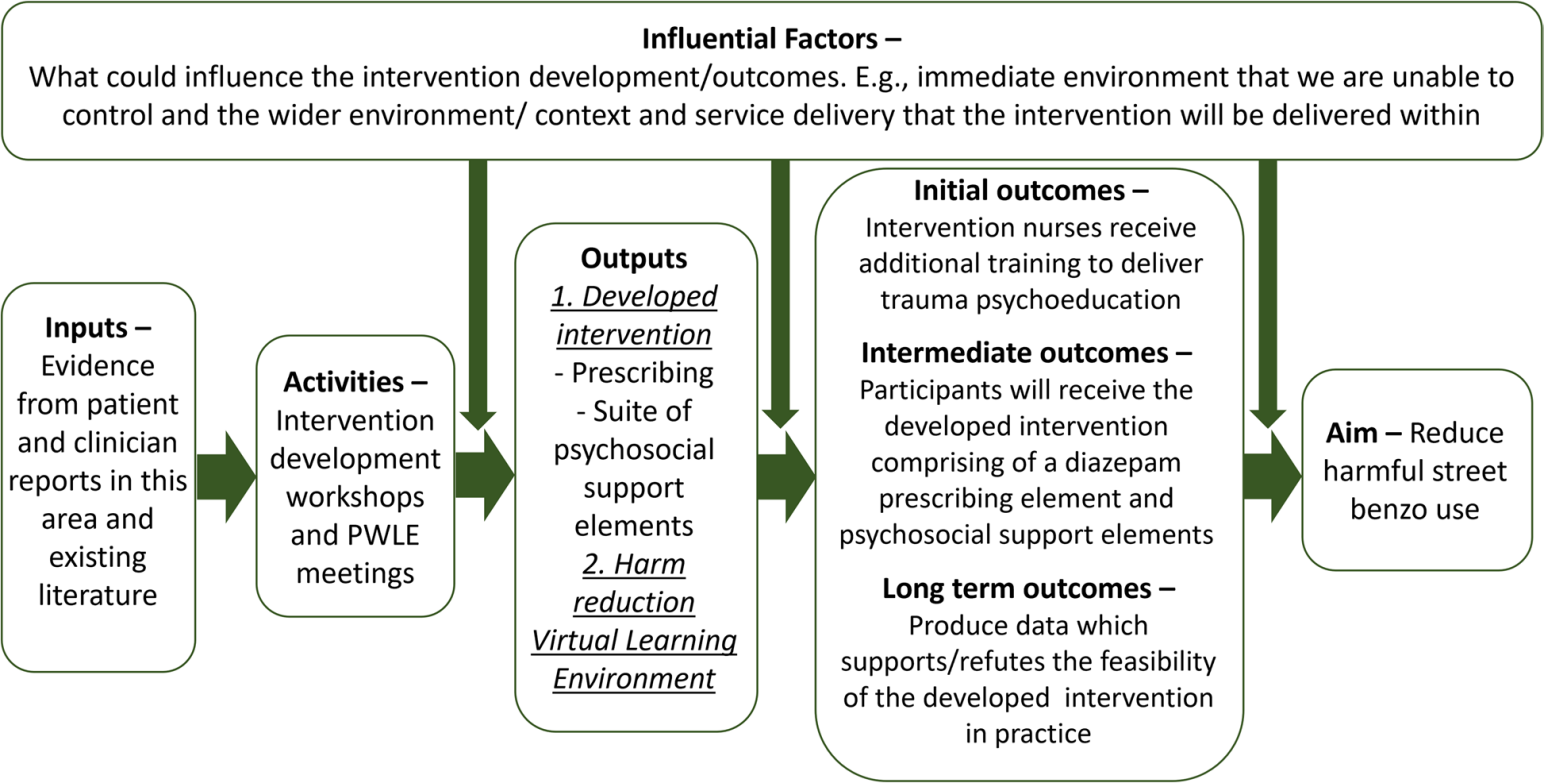
Logic model

At the end of the process, we had been able to produce a completed logic model (Fig. 2: Logic model) for the proposed intervention, as well as finalising the elements of the intervention ahead of feasibility testing Table 4.

**Table 4:** Intervention component summary

Prescribing	Harm Reduction	Psychosocial
• Prescription of diazepam will be provided by the local clinical lead with a maximum dose of 30mg daily, depending on clinical assessment (in line with the Orange Guidelines) [6] • Participants GP and Community Pharmacist will be informed by letter that they are taking part in the study • The prescription will be reviewed monthly between the patient, intervention nurse and the prescriber	• Lockable boxes • Harm reduction advice^a^ • Safety conversations^a^	• Trauma psychoeducation • Anxiety management • Sleep assessment • Pain management • Peer support group

Intervention participants also had access to a bespoke virtual learning environment including links to further information on the above elements of the intervention as well as discussion boards relating to these elements where they can talk to other participants, members of the research team and intervention nurses

^a^Management of high-risk benzodiazepines including ‘megadosing’ was addressed through safety conversations and harm reduction advice. This included discussion on the impact on memory, and the risk of overdose of high dose benzodiazepines alongside other drugs. The dosage of diazepam was set at 30mg max per day and this was accompanied by strong messages about risk of concurrent use

## Discussion

The development of this complex intervention was driven by several factors including the rise in drug related deaths in Scotland implicating both benzodiazepines and opiates [3], as well as a lack of flexibility in the provision of prescribed benzodiazepines to patients receiving ORT in the existing treatment system due to a lack of supportive clinical guidance. It was important that any intervention developed placed the needs and motivations of people who use benzodiazepines at its centre. In addition, because of the conflicting nature of the evidence surrounding benzodiazepine prescribing, the intervention needed to be acceptable to clinicians prescribing to this complex patient group. From the outset we incorporated both the MRC framework for complex interventions which describes an approach which considers broader systems awareness [20]. Furthermore, the process was informed by the taxonomy of approaches to developing interventions to improve health [21].

The European Monitoring Centre for Drugs and Drug Addictions (EMCDDA) identified the need to ‘regularly review the provision of services available and adapt existing interventions or develop new ones to meet changing needs’ [26]. The level of DRD in Scotland illustrates these changing needs as well as the complexity of the problem when thinking about the patterns of polydrug use and their implication in those deaths. This report highlights the need for those using drugs to be consulted to understand specific drug use problems and its context. Preparatory work for this study included qualitative interviews with people with lived experience of benzodiazepine use to explore their motivations for use [18] and a clinician survey exploring the issues around prescribing benzodiazepines [19]. Bringing these two perspectives, people who use drugs and clinicians, along with researchers, GPs, and psychologists and pharmacists provided the basis for the co-production approach to our intervention development. Further, the intervention development process reported here used co-production [21]. Central to co-production is the active involvement of the target patient group throughout the development process, not just in a way that seeks their views but in a meaningful way that allowed them to influence the process and resulting model for the intervention. This was achieved by seeking the PWLE group’s feedback throughout the process and incorporating that into subsequent iterations of the developing intervention. This is seen to produce more effective interventions with higher acceptability in the target population [27, 28].

There is increasing, but not conclusive, evidence from epidemiological studies across the world and including Scotland that co-prescribing ORT and benzodiazepine has detrimental effects through increased risk of mortality and DRD specifically [10]. However, the same body of evidence also tends to demonstrate increased retention in treatment for those co-prescribed. Keeping people in treatment longer allows more time for some of the more involved psychosocial and harm-reduction based components of a complex intervention to be delivered. Epidemiological prescribing studies are particularly subject to bias as those being prescribed benzodiazepines may well have more complex mental health needs. Thus, a controlled trial, which accounts for baseline differences is required. This intervention development process was a first step towards such a trial should the next stage feasibility study be successful. The feasibility study underway is testing the measurement of outcomes (street benzodiazepine use as well as retention in treatment, anxiety, depression, and quality of life) as well as the experience of participants.

## Conclusions

A finalised, agreed logic model for the intervention was achieved that was acceptable from a clinical and people with a lived experience perspective. Key components of the intervention are prescribing of diazepam, anxiety, sleep and pain management and harm reduction resources. This intervention will now be the subject of clinical feasibility testing with a view to a further controlled, adequately powered, clinical trial of effectiveness. Clinical feasibility testing will involve undertaking a small non-randomised study to test various aspects of the intervention. This will include exploring the acceptability of the intervention from a clinical and patient perspective, as well as the feasibility of recruiting sites to deliver the intervention and recruitment and retention in the intervention of those who receive the intervention itself.

### Supplementary Information

**Additional file 1: Appendix 1.** TIDieR for Benzodiazepine Intervention Development Study.

## Data Availability

The datasets generated and/or analysed during the current study are not publicly available due to the protection of the privacy and confidentiality of the participants but are available from the corresponding author on reasonable request.

## Abbreviations

DRD: Drug related deaths
ORT: Opiate Replacement Therapy
EMCDDA: European Monitoring Centre for Drugs and Drug Addictions
PWLE: People with lived experience
MRC: Medical Research Council
CPN: Community Psychiatric Nurses
GP: General Practitioner

## Footnotes

1 . The intervention development process took a total of 6 months and was completed in October 2021. Recruitment to the feasibility study began in June 2022 and data collection ended in June 2023. The study will be completed in August 2023.
