# Supplementary material for: Development of an intervention to manage benzodiazepine dependence and high-risk use in the context of escalating drug related deaths in Scotland: an application of the MRC framework
Source: BMC Health Serv Res. 2023 Nov 4;23:1205. doi: 10.1186/s12913-023-10201-7 (PMC10625279; doi:10.1186/s12913-023-10201-7)
Supplement: Supplementary file 1 — Additional file 1: Appendix 1. TIDieR for Benzodiazepine Intervention Development Study. [file 12913_2023_10201_MOESM1_ESM.docx]

# Appendix 1. TIDieR for Benzodiazepine Intervention Development Study

1. **Brief name**

An intervention to manage benzodiazepine dependence and high-risk use in patients who are receiving opiate replacement therapy (ORT)

1. **Why**

Scotland has the highest rate of drug misuse deaths in Europe. It is a feature of deaths in Scotland that people use combinations of drugs which increases the chance of a drug related death. Many deaths involve street benzodiazepines which can contain unknown substances and be of variable strengths.

The theory behind this intervention relates to harm reduction and provides participants with the tools to explore their motivations for using benzodiazepines and addressing those root causes while providing participants with a ‘safe’ benzodiazepine supply through the prescribing of diazepam.

This study tests a new intervention, designed by consulting people who use benzodiazepines, clinicians and working with researchers. The intervention is a combination of benzodiazepine prescribing, harm reduction and psychosocial support.

1. **What**

Materials:

Intervention nurses are provided with training materials relating to trauma psychoeducation in this group of patients. These materials have been specifically designed/delivered for this intervention. These training materials are then used in a full day training session with an independent trainer designed to upskill CPNs delivering the intervention to work with patients to explore and address their motivations for their benzodiazepine use.

Participants will be provided with a lock box for their medication. They will also be provided with a prescription for diazepam (a benzodiazepine). They will also be given access to an online platform containing harm reduction advice as well as links and information relating to the psychosocial elements of the intervention.

1. **Procedures**

Participants will undertake clinical assessment and treatment with the intervention nurse for the course of the intervention period.

The intervention has three components: (i) Prescribing, (ii) Safety and harm reduction and (iii) Psychosocial support.

##### Prescribing

Participants are provided with a prescription for Diazepam by the clinical lead or intervention nurse (if they are a nurse prescriber) at each site. The maximum starting dose as part of the intervention is 30mg per day which is dispensed in instalments in line with the participants ORT prescription, [or at the clinical lead/intervention nurse’s discretion if on a monthly ORT regimen e.g. long acting buprenorphine injection] (most likely 2 to 3 times per week). Doses will not be supervised at the pharmacy to allow dosing schedules of 3 or 4 times per day. (Orange Guidelines and DDTF benzo guidance)

The patient medication related goals and experience of treatment will be reviewed monthly by the intervention nurse in liaison with the prescribing clinician to inform prescribing decisions regarding stabilisation/maintenance treatment or moving to a tapering dose-reduction regimen. If the patient wants to move to a tapering regimen, a tapering plan will be agreed between the prescribing clinician and the patient and reviewed at each appointment. Existing templates will be used as a guide (Ashton manual on https://www.benzo.org.uk/manual/)

##### Safety and Harm Reduction

1. *Lockable boxes* will be provided with the first prescription, to all participants and participants requested to keep their benzodiazepines in it. These provide safe storage to protect others in the household and provide an additional step to remind people they are taking their medication. There is the potential to use the box for notes and reminders.
2. *Harm reduction advice* and discussion will be undertaken at each interaction. Harm reduction will focus on reducing the risk of overdose. Information on the risks of polydrug use will be supplemented with tools, tips and suggestions to reduce consumption through trying to take less street benzodiazepines and see how you get on? To buy less at a time? If going out carrying less benzodiazepines. In line with health psychology theory and being mindful of the potential for memory impairment, the messaging needs to constant and repeated regularly. There is an option to reinforce specific messages to individuals by text if the participant has a phone they use regularly. For example, a reminder to collect medication.
3. *Safety conversations* take place as part of the regular and ongoing review. Safety conversations relate specifically to the personal safety of the person attending the pharmacy to collect medication. People can be targeted for their medication as well as being targeted to purchase street benzos. Open conversations will enable solutions to be found. [examples from the PWLE group include, don’t tell people you are being prescribed benzodiazepines, avoiding eye contact with people who may try to buy/sell illicit benzodiazepines, varying time of attendance, nurse collection or pharmacy delivery may be options if an individual is particularly vulnerable]. Relapse prevention skills will be developed for those being targeted to buy street benzodiazepines.

##### Psychosocial Support

This will be provided based on a strong therapeutic relationship with the intervention nurse. The intervention nurse will seek to develop a trusting therapeutic relationship in which people can feel safe to discuss their drug use. This will allow the nurse to consider safety planning, thinking about physical, environment, relationships, emotions, strengths, and resources.

A suite of options will be offered that target the motivations for use as well as addressing the risks associated with street benzodiazepine use alongside those being prescribed, opiates and other substances. The support offered will be offered to those who have identified problems with anxiety, sleep, or pain. Peer support will be offered to all. The timing of psychosocial support will be on a case-by-case basis depending on when the patient is sufficiently stable and ready to engage with additional support.

1. *Trauma psychoeducation* – nurses delivering the intervention to be trained to enhanced practice level. For deep routed trauma a referral pathway if/when the patient feels ready will be offered if available (such services have very limited capacity currently).
2. *Anxiety management* (group or individual depending on preference). Groups will be online.
3. *Sleep* assessment and good sleep hygiene habits - on a one-to-one basis.
4. *Pain management*: increasing awareness of the risk of pain increasing as medicines are withdrawn and how to manage this pain without medication.
5. *Peer support* *group:* An informal peer support group will initially be led by the intervention nurse and offered to all intervention patients in that site. This will be a social space to share experiences and provide mutual support. The group will choose and direct their own topics and activities if they choose to continue with it. Initial groups will be fortnightly.
6. **Who provided**
7. Prescribing

Prescribing will be undertaken by the clinical lead (consultant) in each site or by the intervention nurse if they hold a nurse prescribing qualification.

1. Safety and Harm Reduction

Delivered by the intervention nurse(s) at each site

1. Psychosocial support

Delivered by the intervention nurse(s) who are trained community psychiatric nurse(s) with a degree in mental health nursing.

Intervention nurses receive additional bespoke training to enable them to deliver the *Trauma psychoeducation* aspect of the intervention.

1. **How**

The intervention will be delivered face to face by the intervention nurse(s) at each site. Most of the intervention will be delivered on a on to one basis, however a support group will be offered to all participants on a fortnightly basis. This support group will also be held face to face by the intervention nurse. Additional information and peer support (in the form of discussion boards) can be accessed by participants through an online platform.

1. **Where**

Delivered in addiction service clinics at each site. Intervention nurses will need access to a private clinic room for all patient appointments.

1. **When and How Much**

The intervention is delivered over a 6-month period due to the constraints of the trial (if in practice this could continue as long as required). Participants have an appointment with the intervention nurse once every two weeks and up to once a week in cases deemed necessary by the intervention nurse. Each appointment with the intervention nurse lasts for 30-60 minutes. Additional support group requires participants to attend those sessions in addition to one-to-one appointments with the intervention nurse.

1. **Tailoring**
2. Prescribing

Monthly reviews on participants dose of diazepam will be undertaken. Intervention nurses will discuss the patient’s needs and wants with them before consulting with the lead clinician if any changes to the dose are to be made. These decisions are designed to be patient led.

1. Psychosocial support

There are 5 elements to this component of the intervention. Not all participants receive all elements, but it is tailored by the intervention nurse based on their clinical assessment of a patient’s needs through discussion with the participant. Different elements of the psychosocial component may be undertaken at different stages of the intervention but again the suitability of this is assessed by the intervention nurse.
